# Hematological entities with plasmacytic differentiation: a case report 

**DOI:** 10.1186/s13256-023-04082-x

**Published:** 2023-09-26

**Authors:** Arati Inamdar, Siddharth Bhattacharyya

**Affiliations:** Department of Pathology and Laboratory Medicine, Perelman School of Medicine at the University of Pennsylvania, 3400 Spruce Street, Philadelphia, PA 19104-4238 USA

**Keywords:** Case report, Immunoglobulins, Lymphoma, Next generation sequencing, Plasmacytoma

## Abstract

**Introduction:**

Plasmacytoma, a localized tumor of monoclonal plasma cells without any clinical, radiological or physical evidence of plasma cell neoplasm (PCN), is a rare entity that accounts for 1% of PCN. Immunoglobulin M (IgM) extramedullary plasmacytoma of mediastinal region has never been reported and is a diagnostic challenge considering other differential diagnoses.

**Case presentation:**

We present the case of a 51-year-old African-American female with progressively increasing cough, dyspnea, and dysphagia for 6 months with a computed tomography (CT) scan revealing a subcarinal mass. The histopathological analysis of the mass reveals a diagnosis of lymphoma with plasma cell differentiation, with a differential of lymphoplasmacytic lymphoma and plasma cell neoplasm. The lymphoma panel via next-generation sequencing (NGS) and a myeloma-targeted fluorescent in situ hybridization (FISH) panel confirmed the diagnosis of IgM extramedullary plasmacytoma, an entity of rare occurrence. Treatment with radiation led to complete regression of the plasmacytoma with normal blood work-up.

**Conclusions:**

This report describes the challenges of diagnosing IgM extramedullary plasmactyoma. Our case report highlights the importance of cytogenetics and NGS in establishing a correct diagnosis that indeed has prognostic and therapeutic implications.

**Supplementary Information:**

The online version contains supplementary material available at 10.1186/s13256-023-04082-x.

## Highlights

- Distinguishing IgM plasmacytoma from other IgM-related disorders requires correlation with clinical, laboratory, and radiologic findings.
- Next generation sequencing and Fluorescent in-situ hybridization have diagnostic utility in hematological entities with plasmacytic differentiation.
- IgM plasmacytoma is a relatively curable hematological condition with an excellent prognosis.

**Supplementary Information:**

The online version contains supplementary material available at 10.1186/s13256-023-04082-x.

## Introduction

Plasma cell neoplasms are defined as an expanded monoclonal population of single immunoglobulin (Ig) secreting terminally differentiated B cells. The secreted monoclonal Ig is called an M protein [1]. The term PCN encompasses various entities such as Multiple Myeloma (MM), Plasmacytoma, Monoclonal immunoglobulin deposition diseases, and Plasma cell neoplasm with paraneoplastic syndrome [1]. Histologically, PCNs are composed exclusively of plasma cells arranged in clusters or sheets. Plasmacytomas are mainly of two major types: solitary plasmacytoma of the bone and extraosseous (extramedullary) plasmacytoma. These plasmacytomas are reported to constitute 1% of PCNs and predominantly affect males of median age 55 years [2].

We present a case of extramedullary plasmacytoma presenting as a subcarinal mass in a woman leading to obstructive symptoms. The histopathological examination revealed sheets of plasmacytoid cells with expression of IgM, confounding a final diagnosis. Thus, molecular and cytogenetic testing was performed to elucidate the final diagnosis. This is the first report of Immunoglobulin M (IgM) plasmacytoma of the mediastinal region, underscoring the crucial role of molecular and cytogenetic-based ancillary studies to precisely diagnose a hematological entity with extreme plasma cell differentiation.

## Case presentation

**Fig. 1:**
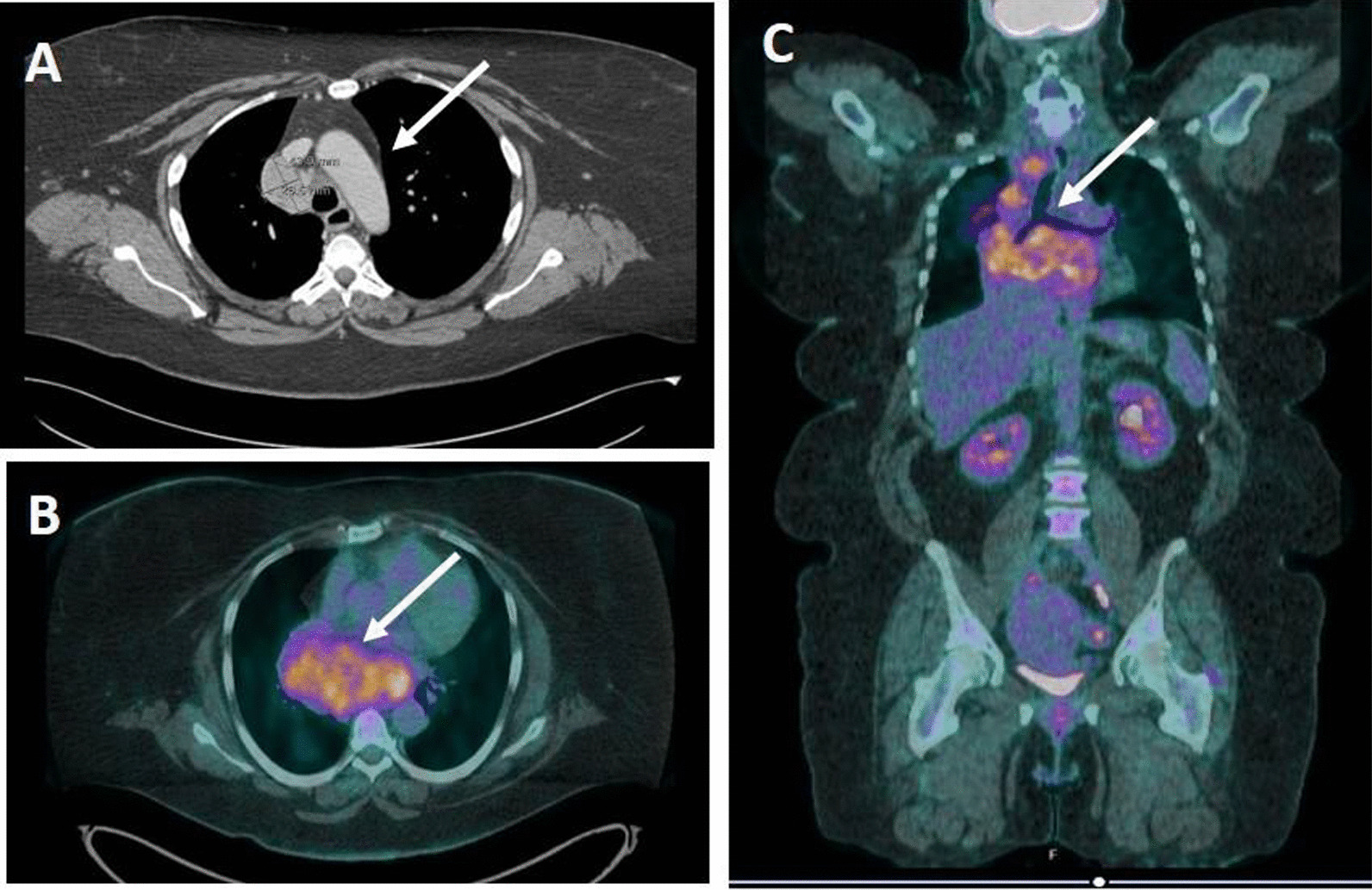
Imaging of the mediastinum mass. **A** Transverse computed tomography image demonstrating the mass (arrow). **B**, **C** Transverse and coronal plane positron emission tomography scan images highlighting the mediastinal mass obstructing the esophagus (arrows)

**Fig. 2:**
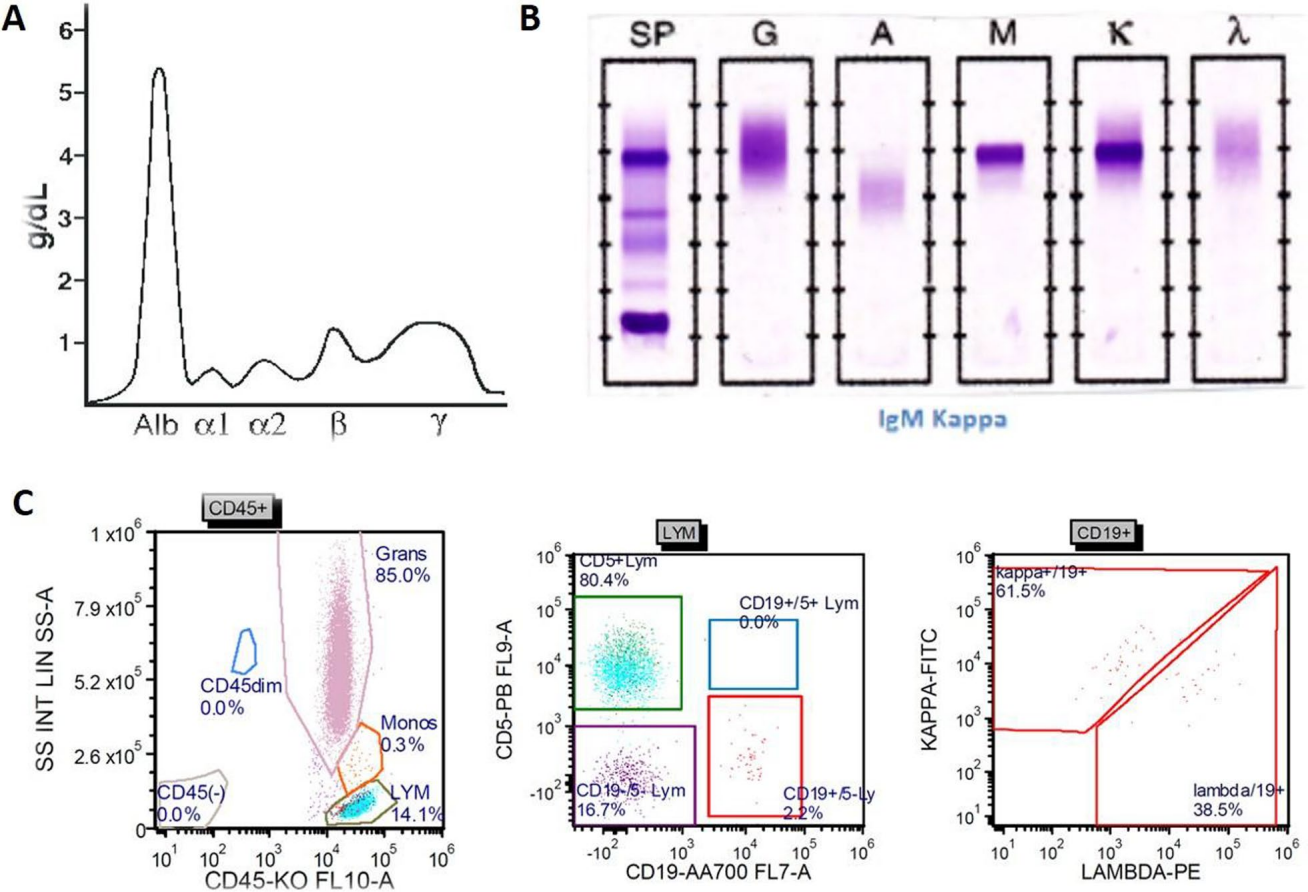
Serum protein electrophoresis (SPEP) and flow cytometry analysis of mass. **A**, **B** The SPEP shows an increase in gamma globulins correlating with an increase in kappa-predominant immunoglobulin M. **C** Flow cytometry analysis of the mediastinal mass, however, failed to detect any immunophenotypic aberrancy

**Fig. 3:**
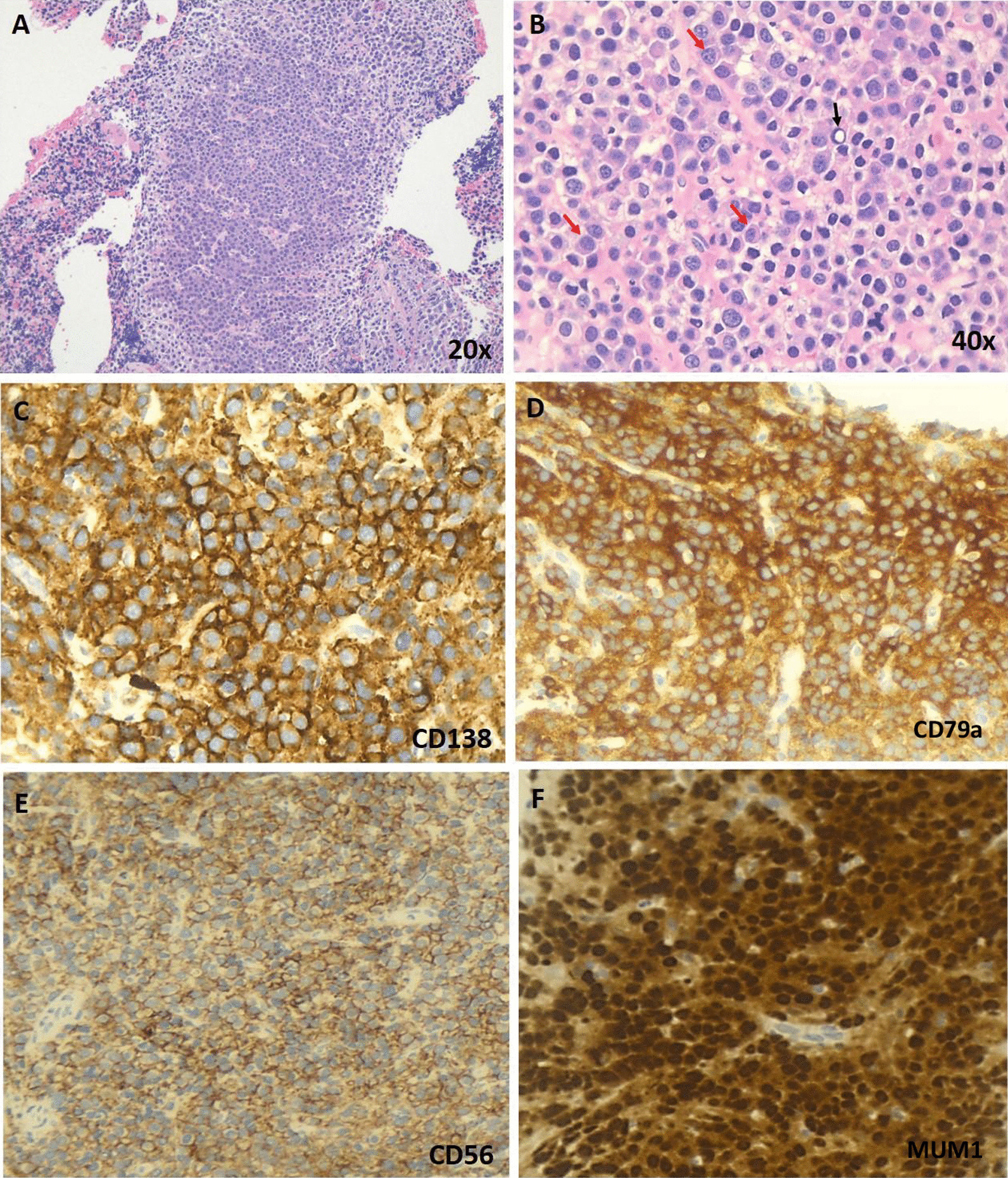
Microscopic description and immunophenotype of mediastinum mass. **A**, **B** The Hematoxylin and Eosin examination of mass showed sheets of lesional cells with oval to round eccentric nuclei and gray-pink cytoplasm (red arrows) with readily evident Ducher bodies (black arrows). The immunohistochemistry performed on the biopsy specimen demonstrated positive staining for CD138 (**C**), CD 79a (**D**), CD56 (**E**) and MUM1 (**F**) confirming the plasmacytic immunophenotype

**Fig. 4:**
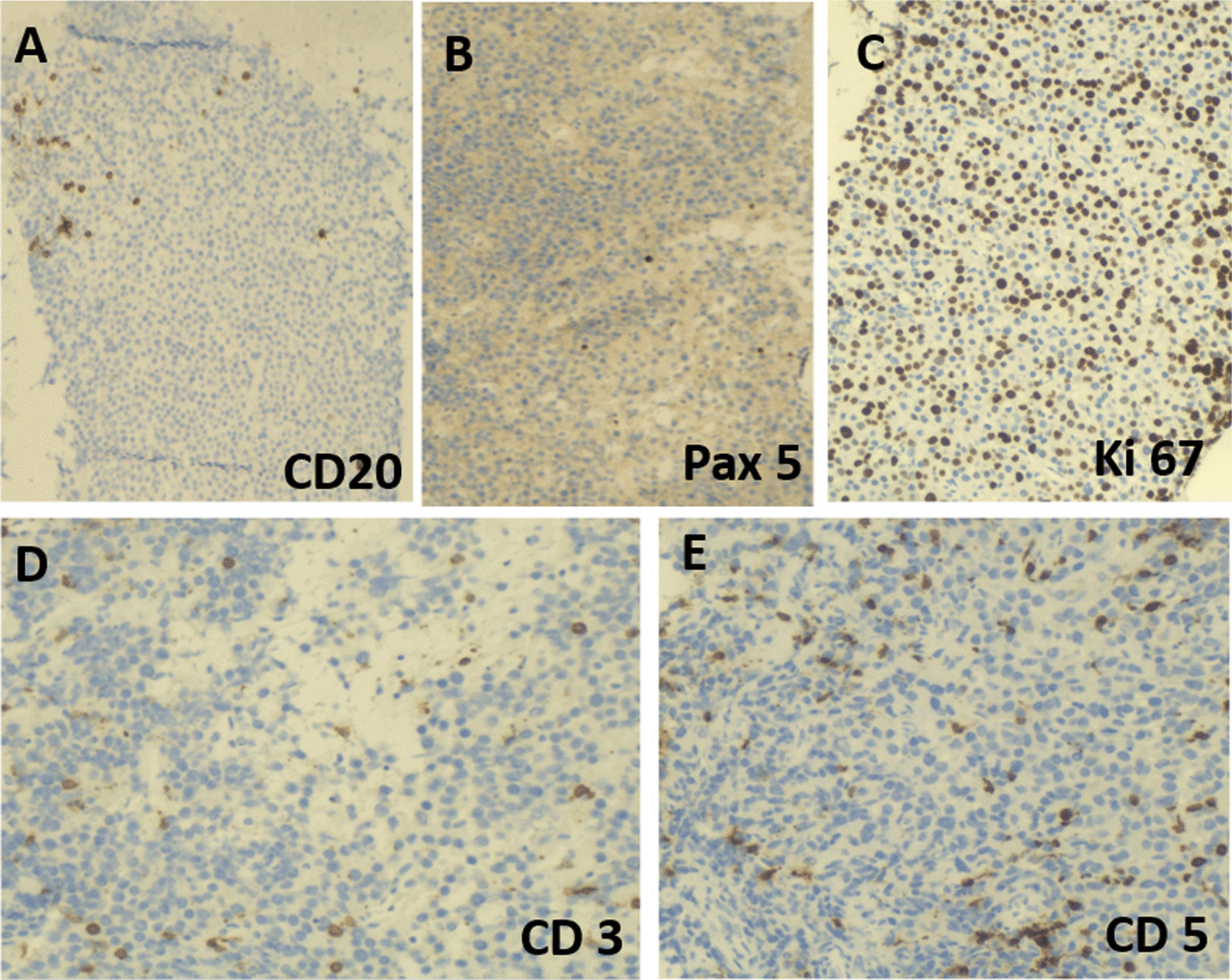
Immunohistochemistry to assess the immunophenotype of mediastinal mass. **A**, **B**, **D**, **E** The lesional cells are negative for CD20, Pax5, CD3, and CD5 with a proliferative index, assessed with Ki67, of 60% (**C**)

**Fig. 5:**
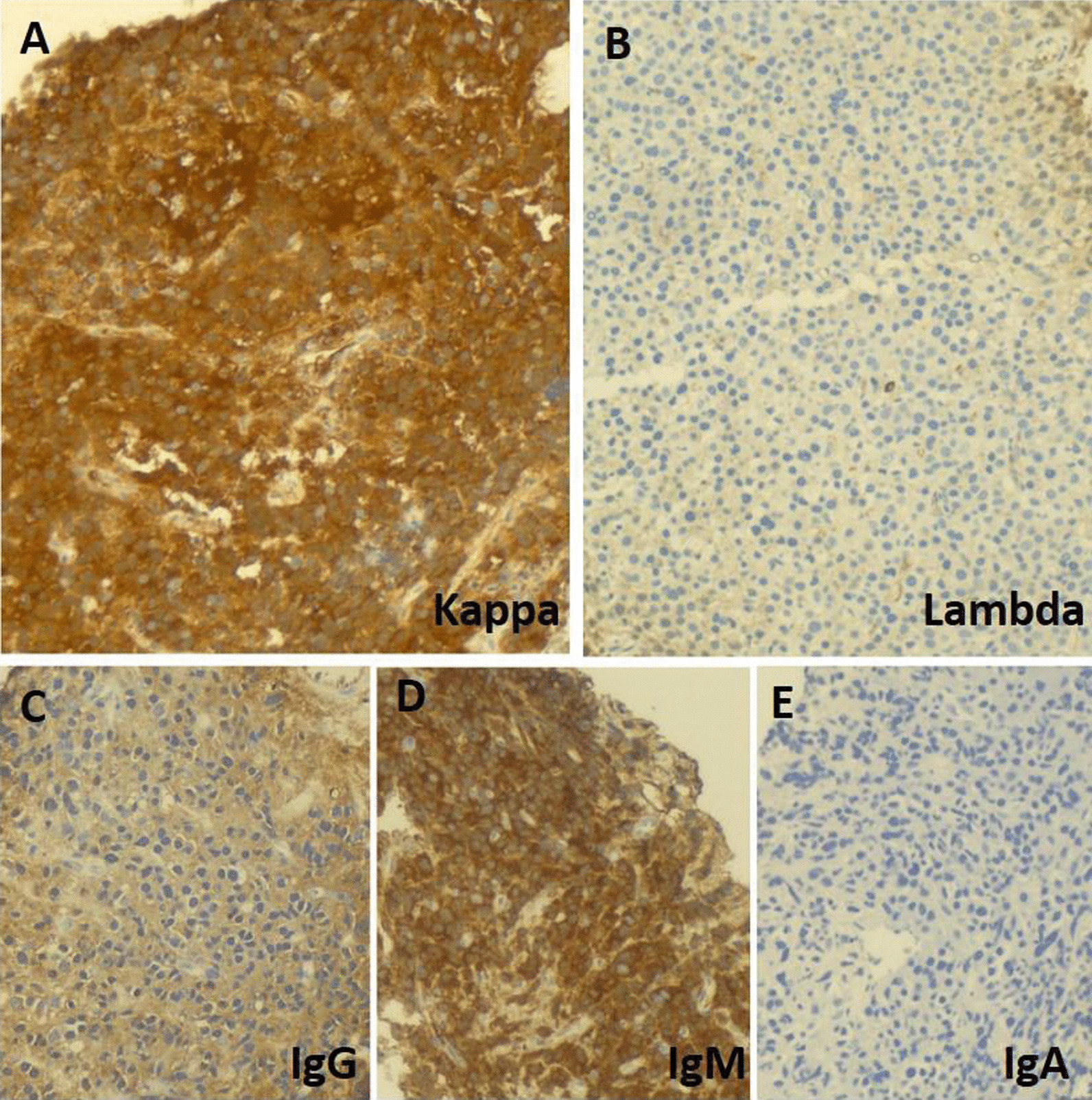
Immunohistochemistry to assess the immunophenotype of mediastinal mass. **A**, **B** The lesional cells demonstrate kappa light chain overexpression alongside increased immunoglobulin M, immunoglobulins with normal immunoglobulin G and immunoglobulin A expression (**C**, **D**, and **E**)

A 51-year-old morbidly obese African-American woman with a 6-month history of progressive voice change, dysphagia, and dyspnea on exertion presented to the clinic. Her past medical history was significant for hypertension, obstructive sleep apnea, gastrointestinal reflux disease, and generalized anxiety disorder. Her social history included a status of a former smoker (1 pack/day for 24 years, quit smoking in 2009) and being married without any biological children. She reports no history of drinking or illicit drug use and has been employed at Tyson Foods as a Manager. No known history of exposure to environmental chemicals. The family history was significant for hypertensive status of both parents. At the time of office visit, her medication included escitalopram, 10 mg tablet orally daily; hydrocholorothiazide 25 mg tablet orally daily, omeprazole 40 mg capsule every morning before breakfast orally and ranitidine 300 mg orally daily. She has been reported to be on these medications for last 5 years. Blood pressure, pulse and temperature were 114/80, 92 and 36.3 °C (97.4 °F), respectively. The overall physical examination was unremarkable except for scattered, intermittent wheezing and prominent vasculature on the chest indicative of Superior Vena Cava (SVC) syndrome. The remaining details of the physical examination are provided in Additional file 1: Table S1. The CBC showed WBC: 12,700/µl; RBC: 5.24 million/µl; Hb:13.3 g/dl; Hct: 41; MCV: 78, MCHC: 25 and platelets: 381,000/µl. The CMP showed glucose: 108 mg/dl; urea nitrogen: 15 mg/dl; creatinine: 0.69 mg/dl; sodium: 138 mmol/l; potassium: 4.2 mmol/l; chloride: 103 mmol/l; carbon dioxide: 26 mol/l and anion gap: 9. The urine analysis was unremarkable. On CT examination, a large, lobulated, heterogenous mass centered in the subcarinal region (10.5 × 6.3 × 6.0 cm) infiltrating the subcarinal and right infrahilar soft tissues as well as the azygoesophageal recess was evident (Fig. 1A). The mass was causing bronchus distortion and right lower lobe collapse. Abnormal air-fluid level in the esophageal lumen causing stasis of contents was evident due to obstruction from the mass. In addition, multifocal mediastinal lymphadenopathy was seen in the right paratracheal, pretracheal and precarinal lymph nodes. This mass demonstrated fluorodeoxyglucose (FDG) activity in the range of 6.5 to 8.5 (Fig. 1B, C). The immunoglobulin quantification showed increased serum IgM (663 mg/dl) with normal levels for IgG and IgA. There was also an increase in kappa light chain concentration to 28.7 mg/L with an increased kappa: lambda ratio of 4.35 by serum protein electrophoresis (SPEP) analysis (Fig. 2A, B). The patient underwent subcarinal lymph node biopsy via endobronchoscopy procedure. On the day of brief hospitalization for this procedure patient continued her routine medications without any changes. The flow cytometry analysis was normal (Fig. 2C). Microscopic examination of the biopsy specimen revealed a lymph node replaced by sheets of lesional cells with oval to round eccentric nuclei and gray-pink cytoplasm with readily evident Dutcher bodies (Fig. 3A, B). The immunohistochemical stains performed on the biopsy specimen showed that the lesional cells demonstrated diffuse positive staining for CD138, CD79a, CD56, and MUM1 (Fig. 3C, D, E, and F) while negative staining for CD20, Pax5, CD5, CD3 with a high Ki67 proliferation index of 60% (Fig. 4A, B, C, D, and E). The immunohistochemical stains for OCT2, CD10, CD30, BCL-2, EBER, BCL 1, CD25, CD45, and CD117 were negative in lesional cells (not shown). These lesional cells demonstrated a kappa-restricted IgM immunophenotype (Fig. 5A, B, C, D, and E). These findings prompted the diagnosis of a clonal lymphoid lineage with plasmacytic differentiation with a differential diagnosis for B-cell lymphoma (i.e. lymphoplasmacytic differentiation vs marginal zone lymphoma) with an extreme level of plasmacytic differentiation and primary de novo IgM plasma cell neoplasm. Cytogenetic studies, karyotyping, FISH studies for PCN, and NGS for lymphoma panel were then performed to further characterized the mass and establish the final diagnosis. The FISH studies revealed an abnormal tetraploid clone with gain of 1q21 and borderline loss of chromosome 17 (*TP53*), while negative for t(4;14), t(11;14), and t(14;16). NGS analysis detected no disease-associated variants or variants of uncertain significance including *MYD88* and *CXCR4.* The final diagnosis was rendered as IgM solitary extramedullary plasmacytoma. The subsequent bone marrow biopsy was normal without any involvement of bone marrow by plasma cell neoplasm. Radiation therapy to the mediastinum (50 Gy) with reassessment after 3 months of treatment demonstrated a reduction in mass. Post-radiation SPEP showed a decrease in IgM levels to 82 mg/dl and free light chain kappa to 10.7 mg/dl with a normal kappa: lambda ratio of 1.51. Three years after the radiation, patient is in complete remission with no interval recurrence with 3 years of follow-up.

## Discussion and conclusions

Our case highlights the diagnostic conundrum associated with diagnosis of IgM solitary plasmacytoma—a rare entity. This is the first report of IgM plasmacytoma of the mediastinal region as IgG based plasma cell neoplasm are most commonly seen. The plasmacytic differentiation seen in lymphoma confounds the diagnosis of such an IgM plasma cell neoplasm/plasmacytoma as lymphoma remains a possible differential diagnosis. The combined approach of morphological, immunohistochemical and molecular testing is necessary in such instances to confirm diagnosis and provide appropriate care and management to patients as inaccurate diagnosis and subsequent treatment may led to increased morbidity and mortality. Our case report highlights the strategic and comprehensive approach towards diagnosis of such hematological lesions with extreme plasmacytic differentiation. During the development of B cells, immature B lymphocytes express IgM as a transmembrane antigen receptor—a part of the B-cell receptor (BCR). B cell activation in lymph nodes in response to antigen binding to the BCR results in rapid cell division and clonal expansion of the activated B lymphocyte, producing many progeny cells. These activated B cells, upon exposure to specific signaling molecules via their CD40 and cytokine receptors (both modulated by T helper cells), undergo antibody class switching to produce IgG, IgA, or IgE antibodies [3]. Memory B cells and plasma cells differentiate from these activated B cells and express somatically hypermutated, high-affinity BCRs of switched isotypes. After exiting the germinal center, these cells secrete class-switched antibodies, predominantly IgG, IgA, or IgE. Hence, IgM-secreting plasma cells are rare and occur in the aberrant absence of class switching or due to arrested development [4]. Clonal populations of these IgM plasma cells constitute an IgM plasmacytoma. According to the WHO, the incidence of plasmacytoma is 1% of PCN. From population-based plasmacytoma incidence and survival data assessed from 12 SEER population-based cancer registries (SEER-12) from1992 to 2004 (SEER-12 2007), the incidence of osseous and extramedullary plasmacytoma was 0.6% and 0.1%, respectively [2]. The PubMed search for “IgM plasmacytoma” reports a handful of cases which are almost three to four decades old [5–8] where the diagnosis of IgM myeloma was mainly based on the histology and predominance of IgM immunoglobulin in the serum [5, 7]. Furthermore, these reports lack a cytogenetic and/or molecular analysis of the specimen, which, in the current era, is routinely performed as an ancillary study for any hematological malignancy. In more recent reports, plasmacytoma was a secondary finding when concurrent with IgM plasma cell myeloma or multiple myeloma—the latter being the findings focused upon in reports [9–12]. Some of these reports have implemented molecular modalities to confirm the diagnosis [12].

The focus of our case is to elucidate a comprehensive approach to diagnose a hematological entity exhibiting the histological features of plasmacytic differentiation where lymphoplasmacytic lymphoma, marginal zone lymphoma, and plasmacytoma are the main differential diagnoses. The importance of distinguishing plasmacytoma from LPL and marginal zone lymphoma is crucial due to differing prognoses and therapeutic options [1].

LPL is an extremely rare neoplasm with an annual incidence of 3 to 4 cases per million people. It has a male predilection with a median age of 70 years. The neoplastic B cell clone is believed to originate from cells at a late stage of B-cell differentiation due to a B-cell arrest after somatic hypermutation in the germinal center and before terminal differentiation to a plasma cell. The neoplastic B cells are associated with IgM monoclonal gammopathy along with visual and neurological symptoms, which are related to hyperviscosity and sluggishness of blood flow. Histologically, the sheets of small neoplastic cells often show variable levels of plasmacytic differentiation and usually show immunoglobulin gene rearrangement and somatic hypermutation with an *MYD88 L265P* mutation, and other less common mutations, namely *CXCR4*, *ARID1A*, *TP53*, *CD79B*, *KMT2D*, and *MYBBP1A* [13]. The presence of *MYD88 L265P* mutation is not specific to LPL and can also be found in non-germinal center subtype diffuse large B cell lymphoma (DLBCL), primary cutaneous DLBCL, leg type DLBCL, primary central nervous system DLBCL, and testicular DLBCL [14, 15]. LPL is usually considered an indolent disease with a median survival of 5–10 years and is treated with anti-CD20 antibody and Bruton tyrosine kinase inhibitors [13].

On the other hand, marginal zone lymphomas are less rare neoplasms with an annual incidence of 19.6 per million people. It has a female predilection with a median of 60 years. The neoplastic B cell cell clone originates from the perifollicular marginal zone [16]. There are three recognized subtypes: nodal, extranodal, and splenic. Each of these show a histology of monomorphic small lymphocytes with variable levels of plasma cell differentiation. Patients have asymptomatic, localized, or disseminated disease with or without lymphadenopathy [1]. Of particular interest, plasmacytic differentiation is found in 1/3 cases of MZL. Such differentiation is restricted to light chain secreting plasma cells located in the interfollicular and perifollicular regions. Differentiation can reach up to 80% of neoplastic cells. Cytogenetically, MZLs are characterized by trisomies of chromosomes 3 and 18, and deletion at 6q23, with *API2/MALT1*, *IGH/MALT1*, or* IGH/BCL10* translocations commonly seen in extranodal type [17]. The treatment of MZL is depended on the subtypes: extranodal, being more common in gastric region, is initially treated with combination antibiotic therapy and proton-pump inhibitors, reserving rituximab, radiation therapy, and surgery for non-responders. Nodal and splenic subtypes are treated with rituximab, chemotherapy, and radiation depending on the clinical symptoms and disease status [18]. Plasmacytoma is characterized by clonal plasma cells, normal bone marrow, normal skeletal survey, and no end-organ damage. Plasma cell myeloma or multiple myeloma on the other hand is characterized by clonal plasma cells with the presence of M protein and involvement of bone marrow along with features of end-organ damage [1]. Plasmacytomas often demonstrate the cytogenetic abnormalities commonly seen in multiple myeloma including translocations involving chromosome 14q and trisomies of odd-numbered chromosomes, as well as secondary abnormalities, such as the deletion of chromosome 17p and amplification of chromosome 1q [19]. The chromosome 1q21 and loss of 17 are proposed to be associated with shorter progression-free survival (PFS) and shorter overall survival (OS) [19]. The treatment modality for both osseous and extra-medullary plasmacytoma includes fractionated radiotherapy with a dose of 40–50 Gy over a duration of 4 weeks with an 80% complete remission. Chemotherapy may be utilized if clinically needed [20].

In our patient, despite demonstrating the immunophenotype common to LPL/MZL, the presence of an abnormal tetraploid clone with gain of 1q21 and borderline loss of chromosome 17 (*TP53*) without t(4;14), t(11;14), and t(14;16) and absence of aberrancy in MYD88 L265P or other genes helped in confirming the diagnosis of IgM plasmacytoma. Treatment with radiotherapy led to complete regression of the mediastinal mass and restored a normal K/L ratio and IgM levels without any recurrence within 3 years of follow-up.

The plasmacytic differentiation seen in lymphoma poses a challenge in the diagnosis of a hematological entity with plasmacytic differentiation and can occur in almost all small B cell lymphomas. Hence, although this phenomenon is present commonly in lymphoplasmacytic lymphoma and marginal zone lymphoma, this differentiation is not specific to a certain type of B cell lymphoma and can be seen in other B cell lymphoma such as follicular lymphoma, small lymphocytic lymphoma/chronic lymphocytic leukemia, and mantle cell lymphoma [21]. Thus, the categorization of a hematological entity should be based on the type of lymphoid cells present as well as the morphologic, phenotypic, and molecular/cytogenetic features and clinical presentation. This report describes the challenges of diagnosing IgM plasmactyoma given the various alternative diagnoses. A common histopathology of plasmacytic differentiation in the presence of increased IgM immunoglobulin makes distinguishing between MZL, LPL, and plasmactyoma difficult. Our case report highlights the importance of cytogenetics and NGS in establishing a correct diagnosis, which has significant prognostic and therapeutic implications.

### Supplementary Information

**Additional file 1: Table S1.** The details of physical examination of the patient at the time of admission.

## Data Availability

No data was used for the research described in the article.
