# Supplementary material for: Hematological entities with plasmacytic differentiation: a case report
Source: J Med Case Rep. 2023 Sep 26;17:429. doi: 10.1186/s13256-023-04082-x (PMC10521441; doi:10.1186/s13256-023-04082-x)
Supplement: Supplementary file 1 — Additional file 1: Table S1. The details of physical examination of the patient at the time of admission. [file 13256_2023_4082_MOESM1_ESM.docx]

**Table S1.** The details of physical examination at the time of admission.

Constitutional:

   Appearance: Normal appearance.

HENT:

   Head: Normocephalic and atraumatic.

   Mouth/Throat:

   Mouth: Mucous membranes are moist.

Eyes:

   Conjunctiva/sclera: Conjunctivae normal.

   Pupils: Pupils are equal, round, and reactive to light.

Cardiovascular:

   Rate and Rhythm: Normal rate and regular rhythm.

   Pulses: Normal pulses.

   Heart sounds: Normal heart sounds.

Pulmonary:

   Effort: Pulmonary effort is normal.

   Breath sounds: Normal breath sounds.

Abdominal:

   General: Abdomen is flat.

   Palpations: Abdomen is soft.

Musculoskeletal:

   General: Normal range of motion.

   Cervical back: Normal range of motion.

Skin:

   General: Skin is warm.

Neurological:

   General: No focal deficit present.

   Mental Status: She is alert and oriented to person, place, and time.

Psychiatric:

   Mood and Affect: Mood normal.

   Behavior: Behavior normal.
